# High-Risk Esophageal GIST: Imaging and Therapeutic Impact on Atypical Metastatic Lesions

**DOI:** 10.3390/diagnostics15141802

**Published:** 2025-07-17

**Authors:** Predrag Sabljak, Aleksandra Djuric-Stefanovic, Miljana Bubanja, Strahinja Odalovic, Nenad Ivanovic, Milica Mitrovic-Jovanovic, Ognjan Skrobic

**Affiliations:** 1 Clinic for Digestive Surgery, University Clinical Centre of Serbia, Koste Todorovica Street, No. 6, 11000 Belgrade, Serbia; 2 Faculty of Medicine, University of Belgrade, Dr Subotica No. 8, 11000 Belgrade, Serbia; 3 Center for Radiology and Magnetic Resonance Imaging, University Clinical Centre of Serbia, Pasterova No. 2, 11000 Belgrade, Serbia; bubanjamiljana97@gmail.com; 4 Center for Nuclear Medicine, University Clinical Centre of Serbia, No. 26 Višegradska Street, 11000 Belgrade, Serbia

**Keywords:** gastrointestinal stromal tumors, esophagus, GIST metastases, tyrosine kinase inhibitors

## Abstract

This case represents a relatively rare localization of a gastrointestinal stromal tumor involving the distal esophagus with a very unusual mode of metastatic spread to the lymph nodes and bone structures. Diagnostic follow-up showed the effect of tyrosine kinase inhibitor therapy on metastatic lesions, highlighting the necessity of comprehensive diagnostic evaluation in patients with atypical presentations and contributing to the improvement of therapeutic strategies in complex clinical scenarios.

**Figure 1:**
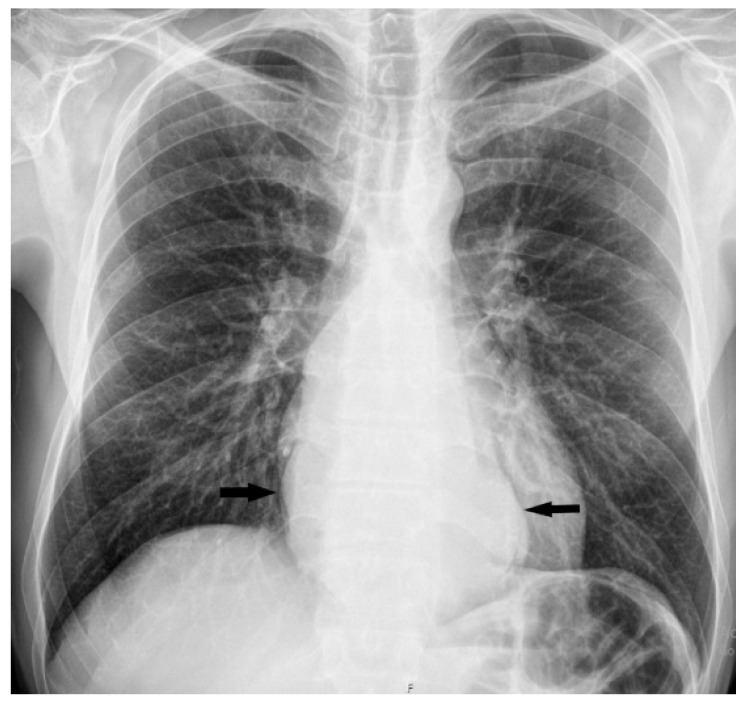
Upright posteroanterior (PA) chest radiograph showed a well-defined, oval soft-tissue opacity located retrocardially (black arrows) in a 45-year-old man who came to our institution with progressive symptoms of dysphagia and anemia, which had been present for about eight months. He presented with Hgb levels of 64 g/L, and his disease history revealed several episodes of hematemesis and melena within the two months prior to admission. Over this period, he unintentionally lost around 20 kg. Esophagogastroduodenoscopy (EGDS) was performed and showed a large, vegetative mass starting at 32 cm from the upper incisors, with almost complete obstruction of the esophageal lumen, though the endoscope could still pass. There was a significant amount of hemorrhagic content inside the lumen of the esophagus and stomach. The mass extended all the way to the cardia, where biopsies were taken. In retroversion, a bulky, oval-shaped polypoid lesion was seen protruding into the gastric lumen from the cardia, roughly 10 cm in size. After EGDS, a multislice CT scan (MDCT) confirmed a large, irregular, partly necrotic mass narrowing the esophageal lumen, spreading caudally across the gastroesophageal junction and infiltrating the cardia and proximal stomach. The tumor was in contact with the pericardium with a lateral impression on the mediastinal pleura, but without radiological signs of infiltration (Figure 2).

**Figure 2:**
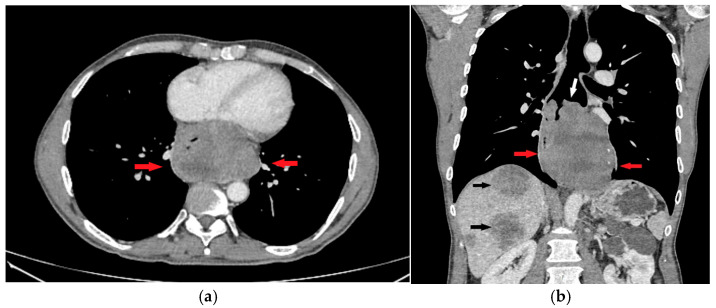
Axial (**a**) CT scan demonstrated an oval, predominantly solid mass with zones of initial necrotic degradation (red arrows), obliterating the esophageal lumen (**b**, white arrow). In liver parenchyma, multiple solid metastatic lesions can be observed (**b**, black arrows). Several enlarged, conglomerated lymph nodes were noted in the celiac, paraaortic, and hepatoduodenal regions.

**Figure 3:**
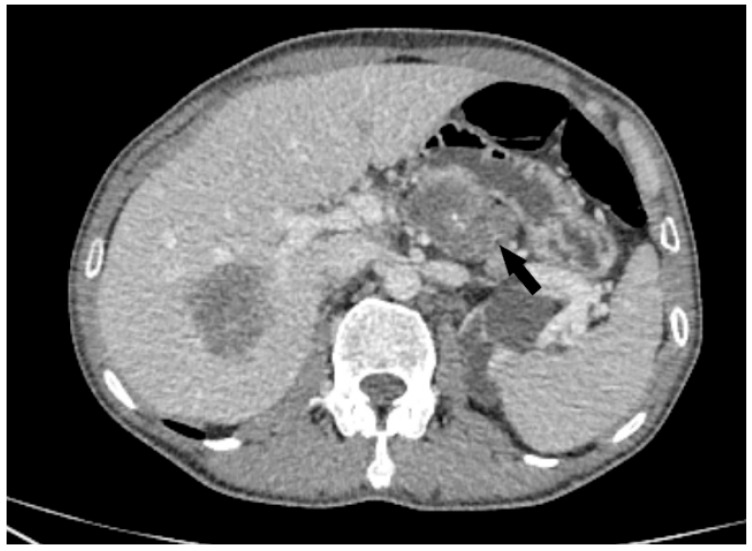
Axial CT image showed a conglomerate of celiac pathological lymph nodes (black arrow) In addition, two solid osteolytic lesions were detected in the sacral and iliac bones on the right, which were highly suspected to be related to the dissemination of the malignant process (Figure 4).

**Figure 4:**
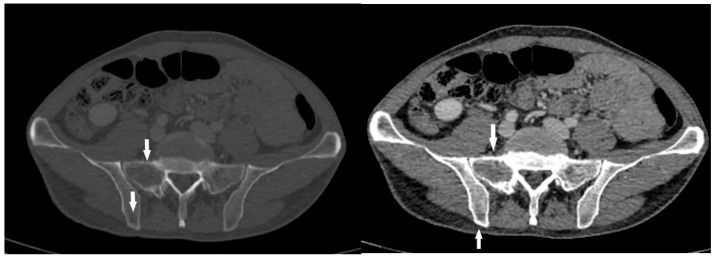
CT axial scan revealed two solid, osteolytic, metastatic lesions (white arrows) in the sacral and iliac bones. Given the imaging characteristics, the possibility of a mesenchymal tumor was considered, although with a very rare and atypical mode of dissemination in the lymph glands and bone structures. After urgent preoperative preparation due to bleeding, the patient underwent a subtotal esophagectomy, with gastric pull-up and intrathoracic esophagogastrostomy performed through a combined left thoraco-phreno-laparotomy. Intraoperatively, a large mass involving the distal esophagus, cardia, and proximal stomach was confirmed. The mass was in close contact with the pericardium and thoracic aorta, but without direct infiltration—it was easily separated from both. The resected specimen included the distal esophagus and proximal stomach. Histopathological and immunohistochemical analysis confirmed a high-risk gastrointestinal stromal tumor (GIST) of the cardia. The tumor was diffusely positive for DOG-1, CD117, and CD34, with a mitotic index of 15/50 HPF (Figure 5). The final diagnosis was pT4 N1 (3/23) M1 (liver and bone), with circumferential margin involvement (R1). Based on these findings, the disease was categorized as stage IV.

**Figure 5:**
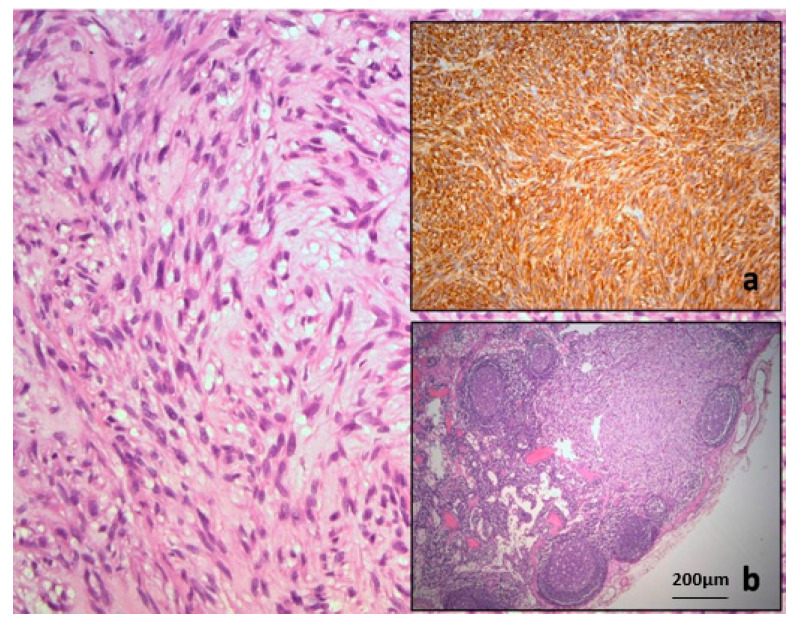
Typical histopathology of gastric gastrointestinal stromal tumor confirmed by strong cytoplasmic immunoexpression of CD117 (**a**). Tumor deposit of GIST found in regional lymph node (**b**). Further, at the first oncology board, systemic treatment with Alvotinib was recommended and initiated. Alvotinib 400 mg (generic imatinib) was administered continuously as the first-line systemic treatment. The patient responded well, and the disease remained stable according to RECIST criteria. Liver metastases showed a satisfactory response to systemic chemotherapy on control imaging (Figure 6 and Figure 7). However, the sacral metastasis remained radiologically stable for two years; after which, we observed increased FDG uptake and dimensional enlargement, consistent with secondary resistance (Figure 8).

**Figure 6:**
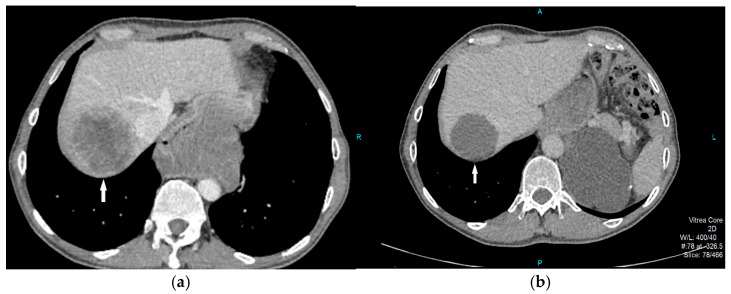
CT axial scans show the preoperative appearance of the metastasis (**a**) in which the solid component of the tumor with a central zone of necrosis dominates (white arrow), while the second image two years later (**b**) clearly shows the positive effect of the implemented therapy, with a predominantly cystically degenerated lesion (white arrow) with dimensional regression of up to 1 cm. R—right, A—anterior, L—left.

**Figure 7:**
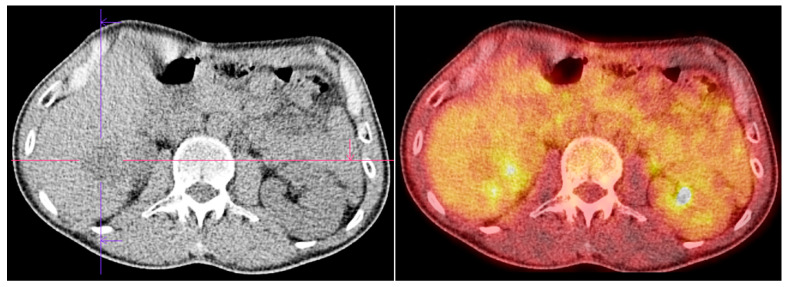
PET/CT shows hypodense liver lesion without significant FDG uptake (SUV max 2.7). There was no abnormal uptake at the esophagogastric anastomosis level, nor in the mediastinal lymph nodes. However, the scan showed a metabolically active, FDG-avid lytic lesion in the right sacral bone, measuring 55 × 65 mm (SUVmax 11.4).

**Figure 8:**
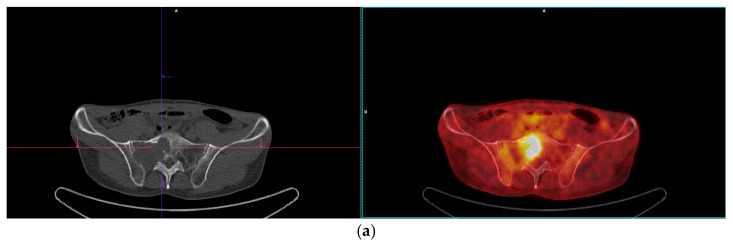

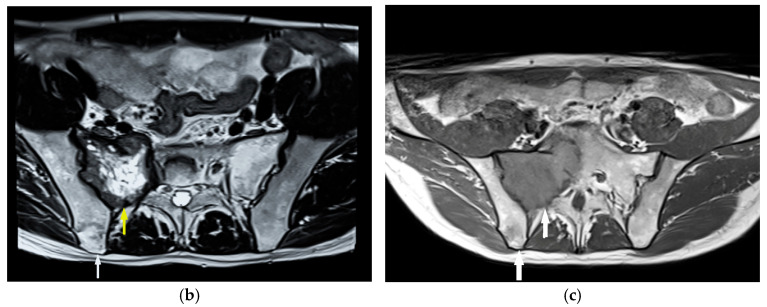
Post-therapy control PET/CT (**a**) and MRI (**b**,**c**) examinations show the progression of metastatic lesions in the sacral and iliac bone. PET scan shows a metabolically active, FDG-avid lytic lesion in the right sacral bone, measuring approximately 55 × 65 mm (SUVmax 11.4, **a**). T2W image (**b**) demonstrates a solid, centrally necrotic, infiltrative lesion with destruction of bone structure and cortex with extraosseal spread. On T1W (**c**), greater extension and dimensional progression of the lesion is observed, indicating an unsatisfactory response to the application of tyrosine kinase inhibitors. At the most recent oncology board following this PET/CT, it was decided that the patient would continue imatinib therapy. Bone metastases in GIST often demonstrate slower metabolic responses to tyrosine kinase inhibitors due to reduced vascular perfusion. However, the parenchymal metastases showed a satisfactory therapeutic response without the appearance of pathological lymph nodes; therefore, the oncology board decided to continue the therapy according to the same protocol, with rigorous monitoring of the sacral bone lesion and the patient’s symptoms. If the progression continues and pain occurs, radiotherapy can be considered as a palliative treatment option. Gastrointestinal stromal tumors (GISTs) are the most common mesenchymal tumors of the gastrointestinal (GI) tract. These tumors originate from interstitial Cajal cells, which are considered the pacemaker cells of intestinal peristalsis. GISTs can develop anywhere along the digestive tract but most commonly arise in the stomach (60%) and small intestine (30%), while duodenal, rectal, esophageal, and colonic forms are less frequent [1]. Although the most prevalent of their type, GISTs are still considered rare tumors, with an annual incidence of approximately 10 cases per million individuals, accounting for 1–3% of all GI malignancies [2]. From an etiological perspective, GISTs most often result from mutations in genes encoding receptor tyrosine kinases—primarily KIT and PDGFRA, whose activation plays a key role in tumorigenesis [3]. For this reason, the introduction of molecularly targeted therapies—particularly imatinib—has significantly improved patient outcomes, especially in the context of metastatic disease [2,4]. In metastatic GIST, the ESMO–EURACAN–GENTURIS guidelines recommend a sequential treatment strategy using successive tyrosine kinase inhibitors. Imatinib remains the foundation of first-line therapy, followed by agents such as sunitinib, regorafenib, and ripretinib in later lines to manage resistance. Whenever possible, molecular profiling should guide the choice of inhibitor, as certain mutations can affect drug sensitivity. Long-term administration of these targeted therapies is crucial since treatment interruption often leads to rapid progression. A multidisciplinary team should also consider local interventions—such as surgery or image-guided ablation—for patients with limited metastatic disease to maximize outcomes [5]. The clinical presentation of a GIST depends on the tumor’s location and size. Patients usually report nonspecific symptoms such as dull abdominal pain, abdominal fullness, loss of appetite, or gastrointestinal bleeding. In some instances, the tumor is incidentally identified during imaging performed for unrelated clinical indications [1]. The liver (50–60%) and peritoneum (20–43%) are the most common sites of GIST metastasis, whereas the involvement of lymph nodes, lungs, and bones is considerably less frequent [6,7]. Atypical metastatic sites, such as lymph nodes and bones, are a diagnostic challenge and may be misinterpreted as unrelated lesions of different etiologies. Rare site metastases may occur in up to 3% of cases, most commonly involving the bones and lungs, and are often associated with poor prognosis [6]. Lymphatic spread is particularly uncommon, and current guidelines do not recommend routine lymphadenectomy unless nodal metastases are suspected based on findings such as enlarged pathological nodes on preoperative CT or increased FDG uptake on PET/CT imaging [8]. Likewise, bone metastases from GISTs are exceptionally rare and have mostly been reported through isolated case studies [9,10]. According to data from the Surveillance, Epidemiology, and End Results (SEER) database, isolated bone metastases occur in only 0.14% of patients, with a median overall survival of just 8 months. Bone metastases are often diagnosed at advanced stages and most frequently involve the spine and pelvis [10]. Clinical symptoms vary based on the affected site, but bone lesions most frequently present with localized pain and may be accompanied by anemia when bone marrow involvement is present [6]. In our case, the simultaneous detection of both skeletal and lymphatic metastases reflects a highly unusual manifestation of the disease and emphasizes the need for thorough diagnostic assessment in patients presenting with atypical symptoms or unexpected radiologic findings. A recent review by Yang et al. highlighted that patients with spinal involvement had significantly poorer outcomes than those without, reinforcing the prognostic importance of metastasis location [11]. The standard therapeutic approach to GIST includes surgical resection as the primary treatment for localized disease, whereas imatinib—a tyrosine kinase inhibitor—remains the first-line therapy for locally advanced and metastatic disease [3,4]. Long-term administration is required, as discontinuation has been associated with rapid disease progression [12]. In cases where patients exhibit disease progression or resistance to the standard 400 mg daily dose, a dose escalation to 800 mg may be warranted—particularly in individuals with KIT exon 9 mutations, who are more likely to benefit from the higher dosage [13]. The effectiveness of imatinib has been demonstrated in multiple clinical trials, showing a clinical benefit in over 80% of patients, with a partial response rate of 53.7% based on RECIST criteria. However, approximately 14% of patients experience early disease progression after initiating therapy, which may reflect the presence of resistant tumor clones [13]. In our patient, hepatic metastases responded favorably to imatinib with no evidence of recurrence, consistent with typical response patterns [4,14]. Interestingly, the sacral bone metastasis exhibited resistance to the same treatment, potentially indicating molecular divergence between metastatic sites or tissue-specific differences in drug absorption and activity [13,14]. Several reports have highlighted variability in imatinib sensitivity between primary tumors and metastatic sites, particularly in those located in bone [15]. This discrepancy further supports the importance of individualized treatment approaches in these patients. Given the potential for early resistance, radiologic monitoring remains essential throughout the course of therapy. PET-CT has proven to be a highly sensitive tool for early assessments of therapeutic responses, although its routine use is limited by availability, and some lesions may not show increased glucose uptake even before therapy initiation [16]. This observation leads to a broader consideration of disease behavior across metastatic sites. This case further supports findings suggesting that the behaviors of GISTs can differ markedly between metastatic locations, underscoring the importance of individual patient assessment [17]. In summary, this case of GIST with synchronous bone and lymph node metastases represents an unusual pattern of dissemination and underscores the biological variability that may exist between different metastatic sites. Such findings highlight the necessity of comprehensive diagnostic evaluation in patients with atypical presentations and may ultimately contribute to the refinement of therapeutic strategies in complex clinical scenarios.

## Data Availability

The datasets used and analyzed in this study are available from the corresponding author upon reasonable request.
